# The effects of several postoperative adjuvant therapies for hepatocellular carcinoma patients with microvascular invasion after curative resection: a systematic review and meta-analysis

**DOI:** 10.1186/s12935-021-01790-6

**Published:** 2021-02-06

**Authors:** Jiarui Yang, Hao Liang, Kunpeng Hu, Zhiyong Xiong, Mingbo Cao, Zhaozhong Zhong, Zhicheng Yao, Meihai Deng

**Affiliations:** 1 Department of Hepatobiliary Surgery, The Third Affiliated Hospital of Sun Yat-Sen University, No. 600, Tianhe Road, Guangzhou, 510530 Guangdong China; 2 Department of General Surgery, Ling Nan Hospital, The Third Affiliated Hospital of Sun Yat-Sen University, No. 2693, Kai Chuang Avenue, Guangzhou, 510530 Guangdong China

**Keywords:** Hepatocellular carcinoma, Prognosis, Postoperative adjuvant transarterial chemoembolization, Postoperative radiotherapy, Radiofrequency ablation, Re-resection, Sorafenib, Microvascular invasion

## Abstract

**Background:**

For patients with hepatocellular carcinoma (HCC) with microvascular invasion (MVI) after curative resection, the effects of various postoperative adjuvant therapies are not summarized in detail, and the comparison between the effects of various adjuvant therapies is still unclear. Thus, we collected existing studies on postoperative adjuvant therapies for patients with HCC with MVI after curative resection and analyzed the effects of various adjuvant therapies.

**Method:**

We collected all studies on postoperative adjuvant therapy for patients with HCC with MVI after curative resection from PubMed, EMBASE, Cochrane Library and SinoMed ending on May 1, 2019. Overall survival (OS) and disease-free/recurrence-free survival (RFS) between each group were compared in these studies by calculating the pooled hazard ratio (HR) and 95% confidence interval (CI). All statistical analyses were assessed by two authors independently.

**Result:**

A total of 13 studies were included in this study, including 824 postoperative adjuvant transarterial chemoembolization (pa-TACE) patients, 90 postoperative radiotherapy patients, 57 radiofrequency ablation (RFA)/re-resection patients, 16 sorafenib patients and 886 postoperative conservative treatment patients. The results showed that pa-TACE significantly improved OS and RFS compared with postoperative conservative treatment in patients with HCC with MVI after curative resection (HR: 0.64, 95% CI: 0.55–0.74, p < 0.001; HR: 0.70, 95% CI: 0.62–0.78, p < 0.001, respectively). There was no significant difference in OS between pa-TACE and radiotherapy in patients with HCC with MVI (HR: 1.75, 95% CI: 0.92–3.32, p = 0.087). RFS in patients with HCC with MVI after pa-TACE was worse than that after postoperative adjuvant radiotherapy (HR: 2.29, 95% CI: 1.43–3.65, p < 0.001). The prognosis of pa-TACE and RFA/re-resection in patients with MVI with recurrent HCC had no significant differences (HR: 0.65, 95% CI: 0.09–4.89, p = 0.671). Adjuvant treatments significantly improved the OS and RFS of patients compared with the postoperative conservative group (HR: 0.580, 95% CI: 0.480–0.710, p < 0.001; HR: 0.630, 95% CI: 0.540–0.740, p < 0.001, respectively).

**Conclusion:**

Compared with postoperative conservative treatment, pa-TACE, postoperative radiotherapy and sorafenib can improve the prognosis of patients with hepatocellular carcinoma with microvascular invasion after curative resection. Postoperative radiotherapy can reduce the recurrence of patients with HCC with MVI after curative resection compared with pa-TACE.

## Background

Liver cancer is the sixth most commonly diagnosed cancer and the fourth leading cause of cancer-related death worldwide [1]. Currently, the main methods to cure liver cancer include surgical resection, ablation and liver transplantation, but the probability of recurrence is still high after curative treatment (5-year recurrence rate reached 70%–80%) [2–4]. As one of many factors affecting the recurrence of HCC, microvascular invasion (MVI) has received extensive attention in recent studies. MVI refers to the HCC microemboli in the portal vein or hepatic vein around the adjacent tumor tissue, which is mainly detected under a microscope and confirmed by postoperative pathology [5]. Existing studies have found that the early recurrence of HCC within 2 years after curative resection is related to the presence of residual micrometastases in the residual liver [6]. As one of the most common residual micrometastases in the liver, MVI plays an important role in the recurrence and survival time of patients with HCC [7]. Many studies have indicated that [8–10] MVI persists in the residual liver after curative resection with a detection rate above 38.7% and is a risk factor for poor prognosis in patients with HCC. Therefore, it is of great significance to find effective therapies for such patients.

According to the existing studies, the major adjuvant therapies for the prevention of postoperative recurrence in patients with HCC with MVI are divided into postoperative adjuvant transarterial chemoembolization (pa-TACE) [11–22], postoperative radiotherapy[12, 13], radiofrequency ablation ablation (RFA) [14] and sorafenib [23]. Pa-TACE is the most common adjuvant therapy after curative resection to effectively reduce the recurrence of HCC [24] and prolong survival time, especially for patients with HCC with portal vein tumor thrombus [25]. However, whether pa-TACE could reduce recurrence and prolong the survival time of patients with MVI is still unclear. With more studies of MVI in the past two years, the application of pa-TACE in the adjuvant therapy of patients with HCC with MVI after curative resection has attracted much attention, but whether it can effectively reduce the recurrence of such patients and prolong the survival time is still controversial. Radiotherapy is an emerging method for the treatment of HCC. For a long time, radiotherapy was not used as a routine treatment in clinical practice due to great damage to the liver. W With the development of radiotherapy technology, an increasing number of studies [26, 27] have found that adjuvant radiotherapy after curative resection could significantly improve the prognosis of patients with HCC. However, the effect of postoperative adjuvant radiotherapy has not been determined for patients with HCC with MVI after curative resection. In addition, further studies are needed to compare the efficacy of postoperative adjuvant radiotherapy with pa-TACE. Ablation, as a radical treatment for HCC, has been widely recognized as a good therapeutic method for patients with single small hepatocellular carcinoma [28–30]. However, its efficacy in the treatment of patients with MVI with postoperative recurrence is rarely reported. Sorafenib is a multitargeted oral drug for HCC. LIovet [31] was the first to show that sorafenib was effective in preventing recurrence and prolonging survival in patients with HCC in 2008. Kim [32] Kim indicated that chemotherapy could better prolong the survival time of patients with HCC with portal venous tumor emboli when compared with sorafenib, but for patients with HCC with postoperative MVI, the effect of sorafenib still needs further research.

We collected and sorted out the current studies on the treatment of patients with HCC with MVI after curative resection and created this systematic review and meta-analysis of the effects of several different treatment methods for such patients.

## Method

Under the guidance of Preferred Reporting Items for Systematic Reviews and Meta-Analyses (PRISMA), we completed this systematic review and meta-analysis [33].

### Criteria for inclusion

We included studies comparing TACE, radiotherapy, ablation, and conservation therapy, all of which met the following inclusion criteria: 1, patients with HCC with MVI, where MVI was diagnosed by postoperative pathology; 2, patients with HCC after curative resection (curative resection: complete resection of the tumor without any tumor tissue at the cutting edge); 3, no other treatment was performed before surgery; 4, no metastatic cancer; 5, no macrovascular invasion; 6, Child–Pugh A-B; 7, outcome included OS and RFS, and full-text studies contained sufficient information or available data for calculating hazard ratios (HRs) with 95.0% confidence intervals (CIs); and 8, randomized double-blind controlled clinical trials (RCTs) and retrospective studies. The exclusion criteria were as follows: 1, patients with HCC without MVI; 2, acceptance of preoperative adjuvant therapy; 3, study without comparison; 4, no available OS or RFS data; and 5, reviews, letters, commentaries and studies published only as abstracts. Table 1 describes the target population, interventions, comparisons and outcome criteria of the study.

**Table 1:** Population, intervention, comparison and outcomes (PICO) of the proposed question:

	Interventions to treat HCC patients with microvascular invasion
Population	HCC patients diagnosed with MVI by pathology after curative resection and Child class A or B without macrovascular invasion and metastatic disease
Intervention vs Comparison	TACE versus conservation therapy; pa-TACE versus re-resection/ radiofrequency ablation ablation; pa-TACE versus radiotherapy; sorafenib versus conservation therapy
Outcomes	OS and/or RFS
Study design	Retrospective study (PSM or not) and randomized clinical trial

*Pa-TACE* postoperative adjuvant transarterial chemoembolization

### Search strategy

We extensively searched all relevant Chinese and English studies ending in May 2019 from multiple databases. These databases included PubMed, Medline, Embase, the Cochrane Library, Web of Science, and SinoMed. The search strategy was designed and executed by two experienced investigators. Differences arising from the search were resolved through negotiation or arbitration by a third examiner. The key search terms were (‘hepatocellular carcinoma’ or ‘liver cancer’ or ‘HCC’) AND (‘MVI’ or ‘microvascular invasion’) AND (‘treatment’ or ‘therapy’). We manually screened other potential studies from the references.

### Data extraction

We extracted the following data from each included study: 1, author name, year of publication and country; 2, basic information of patients, number of patients, age, AFP, BCLC stage, number of males, follow-up time, and Child–Pugh score; 3, details of intervention measures; and 4, HR and 95% confidence intervals for OS and RFS. For literature that did not provide HR values, we used Engauge Digitizer software to extract the HR and its 95% confidence interval for OS and RFS from Kaplan–Meier curves.

### Assessment of methodological quality and risk of bias:

Cochrane risk of bias tools were used to evaluate the methodological quality of RCTs from six aspects, including selection bias, performance bias, detection bias, attrition bias, reporting bias and other bias. These six aspects were divided into low risk, unclear risk and high risk. We used the Newcastle–Ottawa Scale (NOS) to evaluate the methodological quality of retrospective studies [34], Scores over 7 were considered high quality, 4–6 medium quality and less than 4 low quality.

### Data synthesis and statistical methods

This meta-analysis was performed with Stata software (version 12.0; Stata Corporation, College Station, TX, USA). HRs with 95% CIs were calculated to analyze the effect of several different therapies for patients with HCC with MVI after curative resection. Forest plots were applied to exhibit meta-analysis outcomes. The evaluation of statistical heterogeneity was executed by chi-squared tests and I^2^ statistics in this meta-analysis [35]. Heterogeneity was significant when I^2^ > 50% and p < 0.05, and we used a random-effects model. Otherwise, the fixed effects model was adopted. Publication bias was estimated qualitatively using funnel plots with the standard error reported by Egger et al. [36]. Sensitivity analysis was also carried out to confirm the reliability and stability of the results.

## Results

**Fig. 1:**
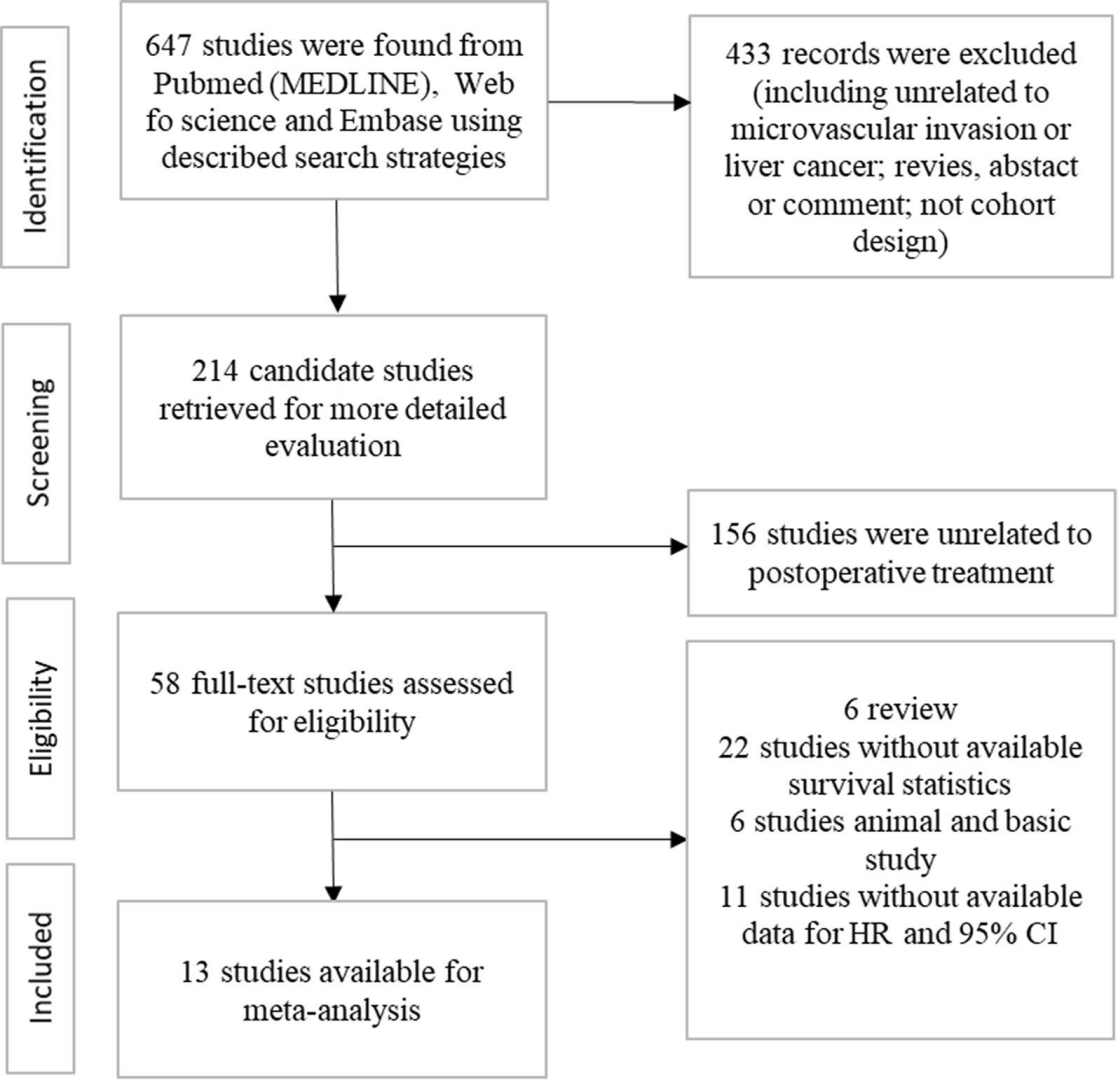
Schematic flow diagram for selection of included studies

A total of 647 studies were initially searched after a series of screenings (Fig. 1). Finally, 13 studies were included by screening the full texts (1 RCT and 12 retrospective studies). A total of 1873 patients were enrolled in this study, including 715 patients with pa-TACE, 90 patients with postoperative radiotherapy, 57 patients with RF/resection, 16 patients with sorafenib and 995 patients with postoperative conservative treatment. All studies were from China. Details of the patient characteristics and treatment methods in the studies are listed in Table 2, and the results are summarized in Table 3.

**Table 2:** Characteristics of the included studies

Author, year	Country	Study design	Variety of analysis	Intervention	Case (n)	BCLC	AFP	Age (year)	Male	Curative resection	Follow up months	Outcome	NOS score
Xiao, 2019 [14]	China	RS (PSM)	MA	Re-resection/RFA	49	BCLC B-C 22	> 200 32.7%	NR	43	yes	84	OS	5
				Pa-TACE: carboplatin 300 mg, epirubicin 50 mg, mytomycin C 8 mg	49	BCLC B-C 25	> 200 38.8%	NR	46	yes	84	OS	
Wang, 2018 [11]	China	RS (PSM)	MA	Conservation	57	BCLC B 10	> 200 40%	56 ± 10	51	yes	60	OS, RFS	7
				Pa-TACE: fluorouracil 750 mg, epirubicin 40 mg or pharmorubici 40 mg	57	BCLC B 11	> 200 44%	55 ± 11	47	yes	60	OS, RFS	
Wang, 2019 [12]	China	RS (PSM)	MA	Radiotherapy: 54–60 Gy	46	NR	> 400 22%	50.98 ± 10.53	43	yes	100	OS, RFS	8
				Pa-TACE: doxorubicin 20–30 mg	46	NR	> 400 15%	51.52 ± 11.40	37	yes	100	OS, RFS	
Wei, 2018 [15]	China	RCT	UA	Conservation	118	NR	≥ 25 69.5%	48.5 (18–74)	106	yes	72	OS. RFS	
				Pa-TACE: carboplatin 200 mg, mitomycin 6 mg, epirubicin 40 mg	116	NR	≥ 25 68.1%	44 (18–75)	106	yes	72	OS, RFS	
Wang, 2018 [16]	China	RS	MA	Conservation	84	NR	367.45 ± 474.58	54.49 ± 10.18	76	yes	84	OS, RFS	8
				Pa-TACE: doxorubicin 10 mg or pharmorubicin 20–40 mg	44	NR	357 ± 444.12	52.07 ± 7.27	42	yes	84	OS, RFS	
Ye, 2017 [17]	China	RS	MA	Conservation	174	BCLC B 47	> 400 48.9%	> 60 19.5%	150	yes	60	OS, RFS	8
				Pa-TACE: lobaplatin 50 mg, raltitrexed 4 mg	86	BCLC B 16	> 400 43%	> 60 15.1%	75	yes	60	OS, RFS	
Wang, 2017 [13]	China	RS	MA	Conservation	50	NR	> 400 36%	57.22 ± 11.14	5	yes	60	OS, RFS	7
				Pa-TACE: adriamycin 20–30 mg	42	NR	> 400 40.5%	51.38 ± 10.89	8	yes	60	OS, RFS	
				Radiotherapy: 54–60 Gy	44	NR	> 400 22.7%	51.32 ± 11.21	5	yes	60	OS, RFS	
Liu, 2016 [18]	China	RS	UA	Conservation	159	BCLC B 12	> 400 57.2%	53 ± 11.8	125	yes	60	OS, RFS	8
				Pa-TACE: fluorouracil 750 mg, epirubicin 40 mg or pharmorubici 40 mg	137	BCLC B 16	> 400 49.6%	49 ± 10.6	134	yes	60	OS, RFS	
Sun, 2015 [19]	China	RS	MA	Conservation	185	BCLC A/B 98	> 400 49.2%	49.91 ± 0.72	167	yes	120	OS, RFS	8
				Pa-TACE: doxorubicin 10 mg or pharmorubicin 20–40 mg	137	BCLC A/b 76	> 400 42.3%	48.88 ± 0.87	120	yes	120	OS, RFS	
Liu, 2016 [20]	China	RS	UA	Conservation	26	NR	NR	NR	NR	yes	40	RFS	5
				Pa-TACE: fluorouracil 1000 mg, oxaliplatin 100 mg, epirubicin 40–60 mg	24	NR	NR	NR	NR	yes	40	RFS	
Jin, 2014 [21]	Korea	RS	UA	Resection/RFA	8	NR	NR	NR	NR	yes	96	OS	5
				Pa-TACE: cisplatin 0.5 mg	23	NR	NR	NR	NR	yes	96	OS	
Huang, 2019 [23]	China	RS	MA	Conservation	33	BCLC B 12.5%	> 400 42.42%	51.52 ± 11.87	30	yes	72	OS, RFS	7
				Sorafenib	16	BCLC B 12.1%	> 400 50%	52.25 ± 11.94	12	yes	72	OS, RFS	
Qi, 2018 [22]	China	RS	UA	Conservation	109	BCLC B 33	> 400 45.8%	NR	93	yes	36	OS, RFS	7
				TACE:lobaplatin (25–100 mg) + pharmorubici (10–50 mg)	91	BCLC B 37	> 400 48.4%	NR	78	yes	36	OS, RFS	

*Pa-TACE* postoperative adjuvant transarterial chemoembolization, *PSM* propensity score matching, *RCT* randomized clinical trial, *RFA* radiofrequency ablation ablation, *MA* multivariate analysis, *UA* univariate analysis, *RS* retrospective study

**Table 3:** Summary of evidence for outcomes of postoperative adjuvant therapies in the included studies

Intervention vs comparison	Design	Studies	Outcome	Patients (n)	ES (95% CI)
Pa-TACE vs conservation therapy	RCT and retrospective study	8	OS	1646	HR 0.64 (0.55–0.74). I^2^ = 4.2%
Pa-TACE vs conservation therapy	RCT and retrospective study	9	RFS	1696	HR 0.70 (0.62–0.78). I^2^ = 0%
Pa-TACE vs radiotherapy	retrospective study (PSM)	2	OS	178	HR 1.75 (0.92–3.32). I^2^ = 0%
Pa-TACE vs radiotherapy	retrospective study (PSM)	2	RFS	178	HR 2.29 (1.43–3.65). I^2^ = 0%
Pa-TACE vs re-resection/ radiofrequency ablation ablation	retrospective study (PSM)	2	OS	129	HR 0.65 (0.09–4.89) p = 0.671, I^2^ = 89.3%
Sorafenib vs conservation therapy	retrospective study	1	OS	49	HR 0.219 (0.071–0.672)
Sorafenib vs conservation therapy	retrospective study	1	RFS	49	HR 0.308 (0.131–0.724)

*Pa-TACE* postoperative adjuvant transarterial chemoembolization, *PSM* propensity score matching, *RCT* randomized double-blind controlled clinical trial

**Fig. 2:**
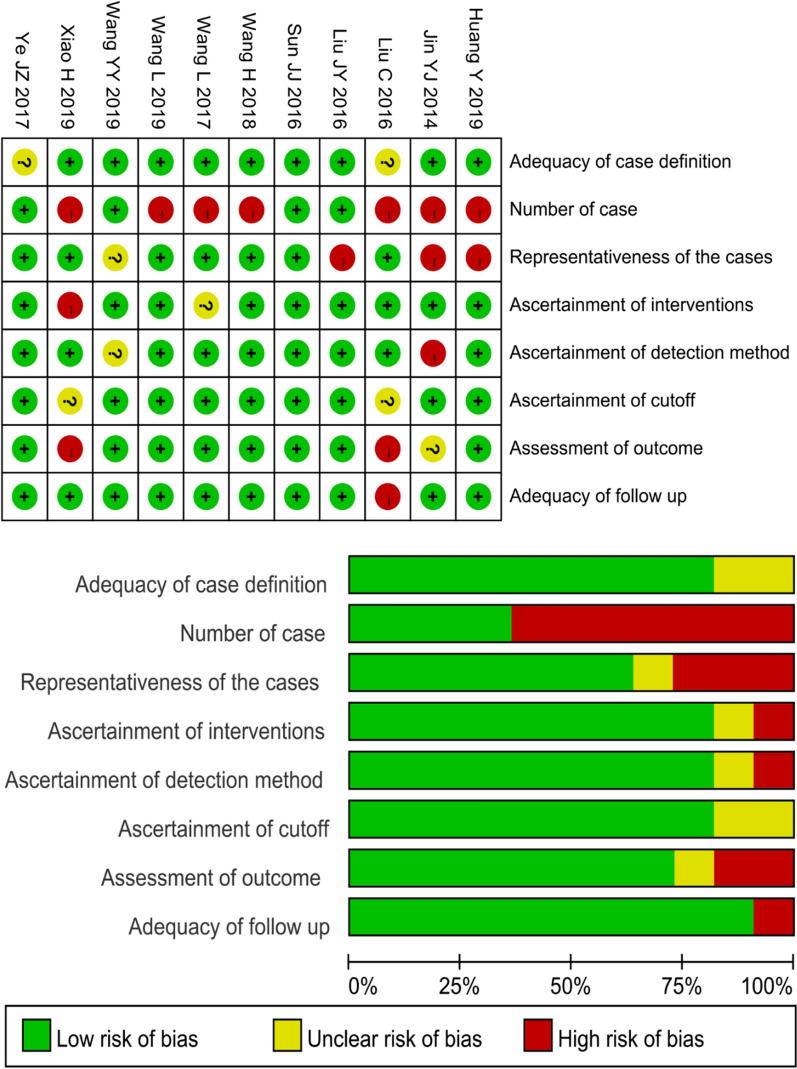
Methodological quality of the retrospective studies

Methodological evaluation of treatment in the included studies:

For the RCT [15], this study adopted the random number allocation method, and sealed and opaque envelopes were used in the allocation process. The blind method was not adopted, but it did not affect the judgment of OS and RFS. Regarding follow-up, the number and reasons for missing patients were similar between the experimental group and the control group. OS and RFS of both groups were reported (Table 4). For retrospective studies, the overall risk of bias was low due to low or unclear risk of adequacy of case definition, follow-up, ascertainment of interventions and detection method, and assessment of outcome. Assessment of the methodological quality for the studies included is reported in Fig. 2 and Table 5.

**Table 4:** Risk of bias assessment of RCT:

	Random sequence generation	Allocation concealment	Blinding of participants and personnel	Blinding of outcome	Incomplete outcome data	Selective reporting	Other bias
Wei, 2018 [15]	Low risk	Unclear risk	Low risk	Low risk	Low risk	Low risk	Low risk

*RCT* randomized clinical trial

**Table 5:** Risk of bias assessment of retrospective studies

	Adequacy of case definition	Case number	Representativeness of the cases	As of interventions	As of detection method	As of cutoff	As of outcome	Adequacy of follow up	NOS score
Huang, 2019 [23]	Low risk	High risk	High risk	Low risk	Low risk	Low risk	Low risk	Low risk	7
Jin, 2014 [21]	Low risk	High risk	High risk	Low risk	High risk	Low risk	Unclear risk	Low risk	5
Liu, 2016 [20]	Unclear risk	High risk	Low risk	Low risk	Low risk	Unclear risk	High risk	High risk	5
Liu, 2016 [18]	Low risk	Low risk	High risk	Low risk	Low risk	Low risk	Low risk	Low risk	8
Sun, 2016 [19]	Low risk	Low risk	Low risk	Low risk	Low risk	Low risk	Low risk	Low risk	8
Wang, 2018 [16]	Low risk	High risk	Low risk	Low risk	Low risk	Low risk	Low risk	Low risk	8
Wang, 2017 [13]	Low risk	High risk	Low risk	Unclear risk	Low risk	Low risk	Low risk	Low risk	7
Wang, 2019 [12]	Low risk	High risk	Low risk	Low risk	Low risk	Low risk	Low risk	Low risk	8
Wang, 2019 [11]	Low risk	Low risk	Unclear risk	Low risk	Unclear risk	Low risk	Low risk	Low risk	7
Xiao, 2019 [14]	Low risk	High risk	Low risk	High risk	Low risk	Unclear risk	High risk	Low risk	5
Ye, 2017 [17]	Unclear risk	Low risk	Low risk	Low risk	Low risk	Low risk	Low risk	Low risk	8
Qi, 2019 [22]	Low risk	Low risk	Low risk	Low risk	Low risk	Low risk	Low risk	Low risk	7

*AS* Ascertainment

### Different OS between pa-TACE and postoperative conservative treatment in patients with MVI

**Fig. 3:**
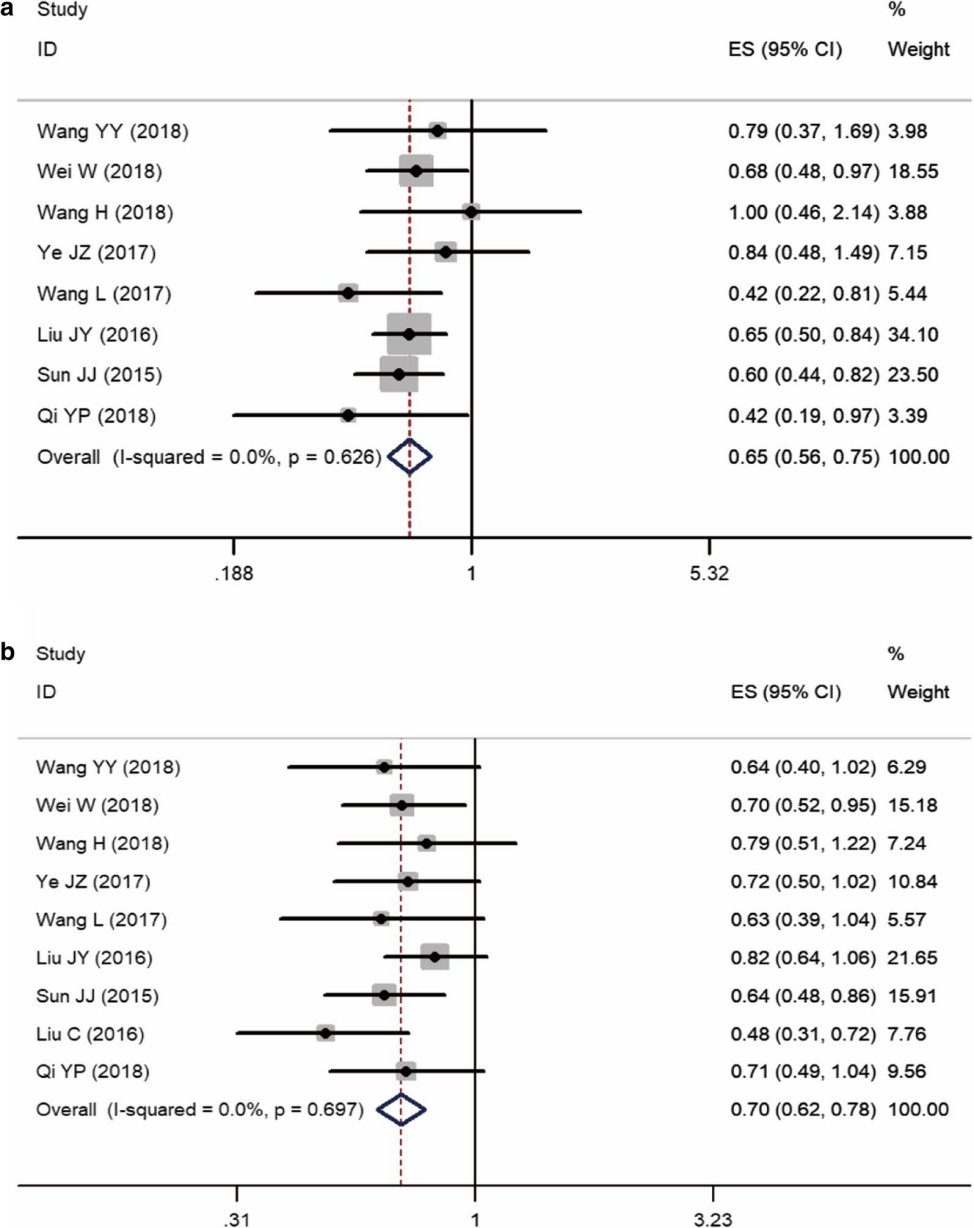
Forest plot of the overall survival and disease-free/recurrence-free survival rates between postoperative TACE and conservation. A overall survival; B disease-free/recurrence-free survival

**Fig. 4:**
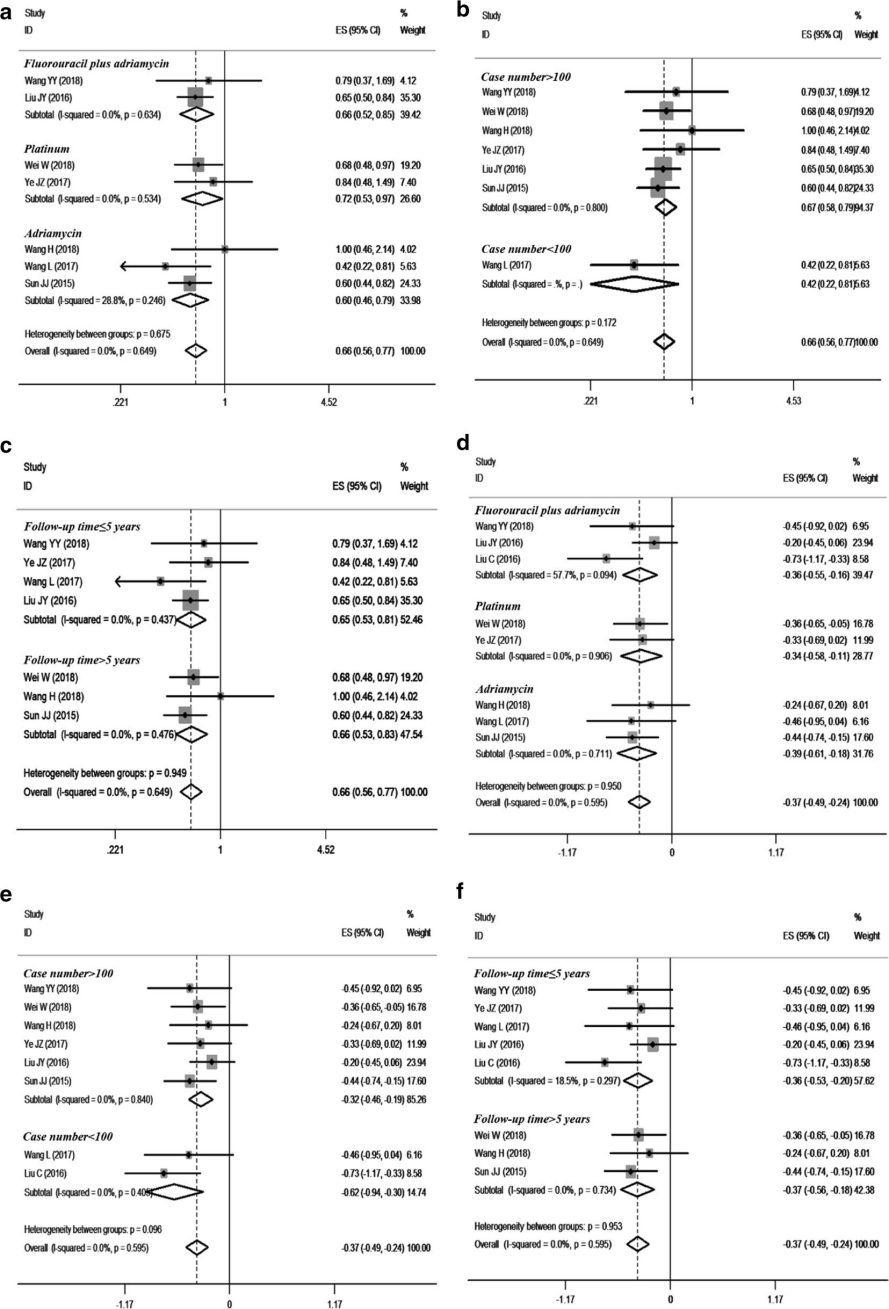
Subgroup analysis of OS and RFS between postoperative adjuvant TACE and conservation. **a **The OS of HCC patients with MVI in three various types of pa-TACE treatment; **b** case number (> 100, < 100), overall survival; **c** follow-up time (> 5 years, ≤ 5 years), overall survival; **d** The RFS of HCC patients with MVI in three various types of pa-TACE treatment; **e** case number (> 100, < 100), RFS; **f** follow-up time (> 5 years, ≤ 5 years), RFS

Eight studies [11, 13, 15–19, 21, 22] c compared the effects of pa-TACE and postoperative conservative treatment on OS in patients with HCC with MVI after curative resection; one was an RCT [15], and the others were retrospective studies [11, 13, 16–19, 21]. Since there was no obvious heterogeneity (I^2^ = 4.2%, P = 0.400), a fixed-effects model was used to analyze the pooled HR. The pooled HR was 0.64 (95% CI: 0.55–0.74, p < 0.001, Fig. 3a). These results suggested that pa-TACE could significantly improve the OS of patients with HCC with MVI after curative resection compared with postoperative conservative treatment. At the same time, we further analyzed the influence of each subgroup on the results. According to the different strategies used in each report, pa-TACE was roughly divided into three categories: fluorouracil combined with doxorubicin (2 studies [11, 18]), platinum (2 studies [15, 17, 21]), and adriamycin (3 studies [13, 16, 19]). The pooled HR of fluorouracil combined with doxorubicin was 0.66 (95% CI: 0.52–0.85, p < 0.001), platinum’s pooled HR was 0.66 (95% CI: 0.56–0.77, p < 0.001), and adriamycin’s HR was 0.60 (95% CI: 0.46–0.79, p < 0.001). Compared with postoperative conservative treatment, all three strategies of pa-TACE significantly improved the OS of patients (Fig. 4a). According to the subgroup analysis of case number, the pooled HR of case number less than 100 (one article [13, 21]) was 0.42 (95% CI: 0.22–0.81, p = 0.009), and the pooled HR of case number more than 100 (6 articles [11, 15–17, 19, 21]) was 0.67 (95% CI: 0.58–0.79). Compared with postoperative conservative treatment, pa-TACE in both groups significantly prolonged the OS of patients (Fig. 4b). In the follow-up time subgroup analysis, three cases were followed up for more than 5 years [15, 16, 19, 21], four cases were followed up for less than 5 years [11, 13, 17, 18], and Pa-TACE in both groups significantly improved the OS of patients compared with postoperative conservative treatment (HR: 0.66, 95% CI: 0.53–0.83, p < 0.001; HR: 0.65, 95% CI: 0.53–0.81, p < 0.001, respectively) (Fig. 4c).

### Different RFS between pa-TACE and postoperative conservative treatment in patients with MVI

Nine studies [11, 13, 15–20, 22] compared the effects of pa-TACE and postoperative conservative treatment on RFS in patients with HCC with MVI after curative resection. One was an RCT [15], and eight were retrospective studies [11, 13, 16–20]. Since there was no significant heterogeneity (I^2^ = 0.0%, P = 0.595), a fixed-effects model was used to analyze the pooled HR. The pooled HR of pa-TACE compared with conservation treatment was 0.69 (95% CI: 0.61–0.78, p < 0.001, Fig. 3b). These results suggested that pa-TACE could significantly improve RFS in patients with HCC with MVI after curative resection compared with postoperative conservative treatment. At the same time, we further analyzed the influence of each subgroup on the results. According to the different TACE strategies used in each report, studies were roughly divided into three categories: fluorouracil combined with doxorubicin (three articles [11, 18, 20]), platinum (two articles [15, 17]), and adriamycin (three articles [13, 16, 19]). The pooled HR of the fluorouracil combined with doxorubicin group was 0.65 (95% CI: 0.47–0.91, p < 0.001), the platinum group’s pooled HR was 0.71 (95% CI: 0.56–0.89, p = 0.003), and the pooled HR of the adriamycin group was 0.67 (95% CI: 0.54–0.84, p < 0.001). Compared with postoperative conservative treatment, all three TACE strategies significantly improved the RFS of patients (Fig. 4d). In the subgroup analysis of case number, the pooled HR of number of cases less than 100 (two articles [13, 20]) was 0.54 (95% CI: 0.39–0.74, p < 0.001), and the pooled HR of number of cases more than 100 (six articles[11, 15–19]) was 0.72 (95% CI: 0.63–0.83). Compared with postoperative conservative treatment, pa-TACE in both groups significantly improved the RFS of patients (Fig. 4e). n the subgroup analysis of follow-up time classification, there were 3 studies [15, 16, 19] with follow-up time over 5 years, five studies [11, 13, 17, 18, 20] with follow-up time less than 5 years, and pa-TACE in both groups significantly improved RFS compared with postoperative conservative treatment (HR: 0.69, 95% CI: 0.57–0.83, p < 0.001; HR: 0.70, 95% CI: 0.59–0.82, p < 0.001, respectively) (Fig. 4f).

**Fig. 5:**
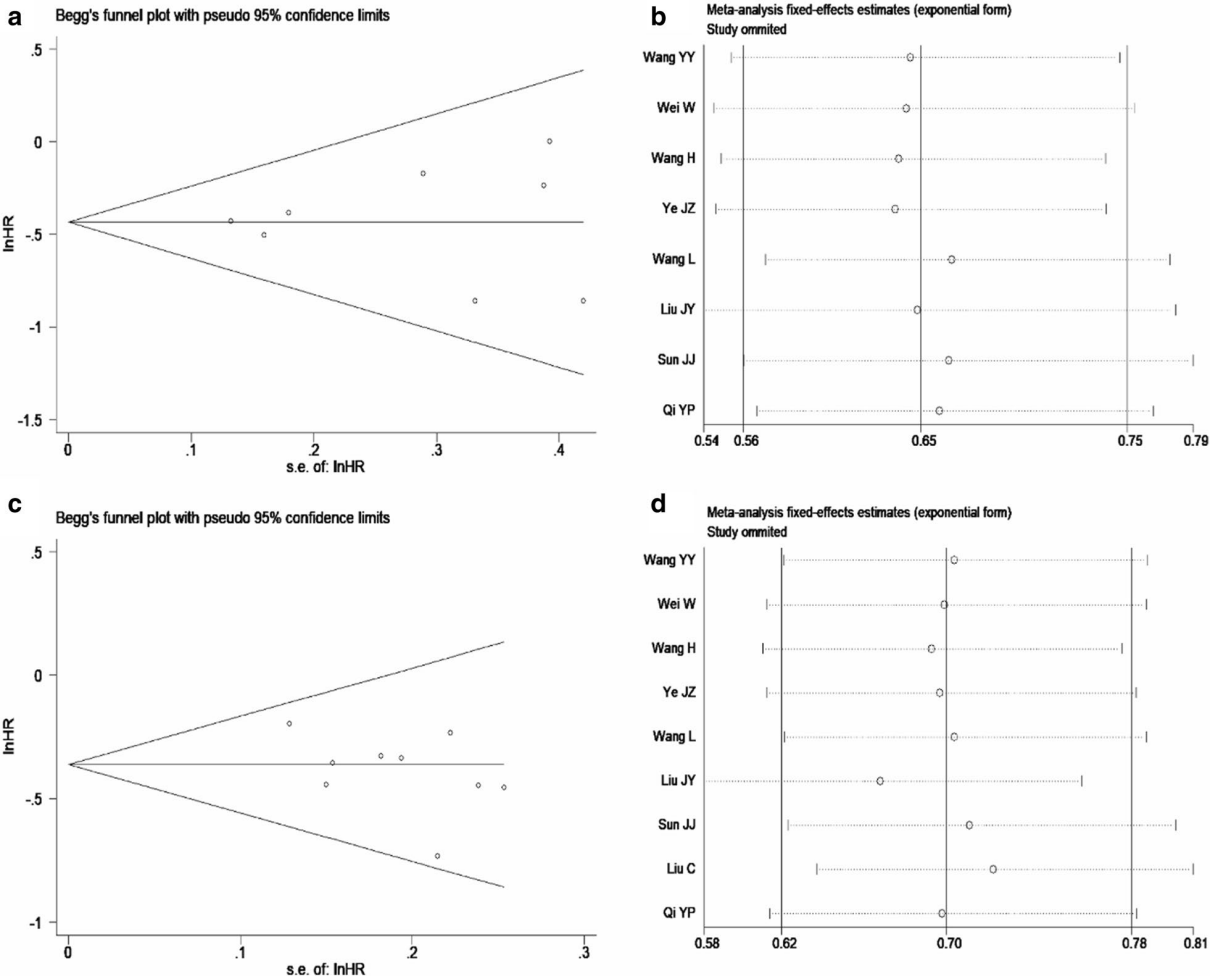
A Begg's funnel plot of the publication bias for postoperative TACE versus conservation in OS of patients, p = 0.711; B Sensitivity analysis of effect of individual studies on postoperative TACE versus conservation in OS of patients; C Begg's funnel plot of the publication bias for postoperative TACE versus conservation in RFS of patients, p = 0.536; D Sensitivity analysis of effect of individual studies on postoperative TACE versus conservation in RFS of patients

A sensitivity analysis was performed by omitting each study in turn from the pooled analysis and calculating the pooled HRs for the remaining studies to determine the influence of each study on the pooled HRs for OS and RFS and confirm the robustness of the results. The results of the sensitivity analysis illuminated that excluding any of the included studies had no significant influence on the final results, and the results of this meta-analysis were relatively robust (Fig. 5). No publication bias was found in Begg's funnel plots of postoperative TACE versus conservation in OS and RFS of patients (the P values were 0.711 and 0.536, respectively).

### Different OS between postoperative radiotherapy and pa-TACE in patients with MVI

**Fig. 6:**
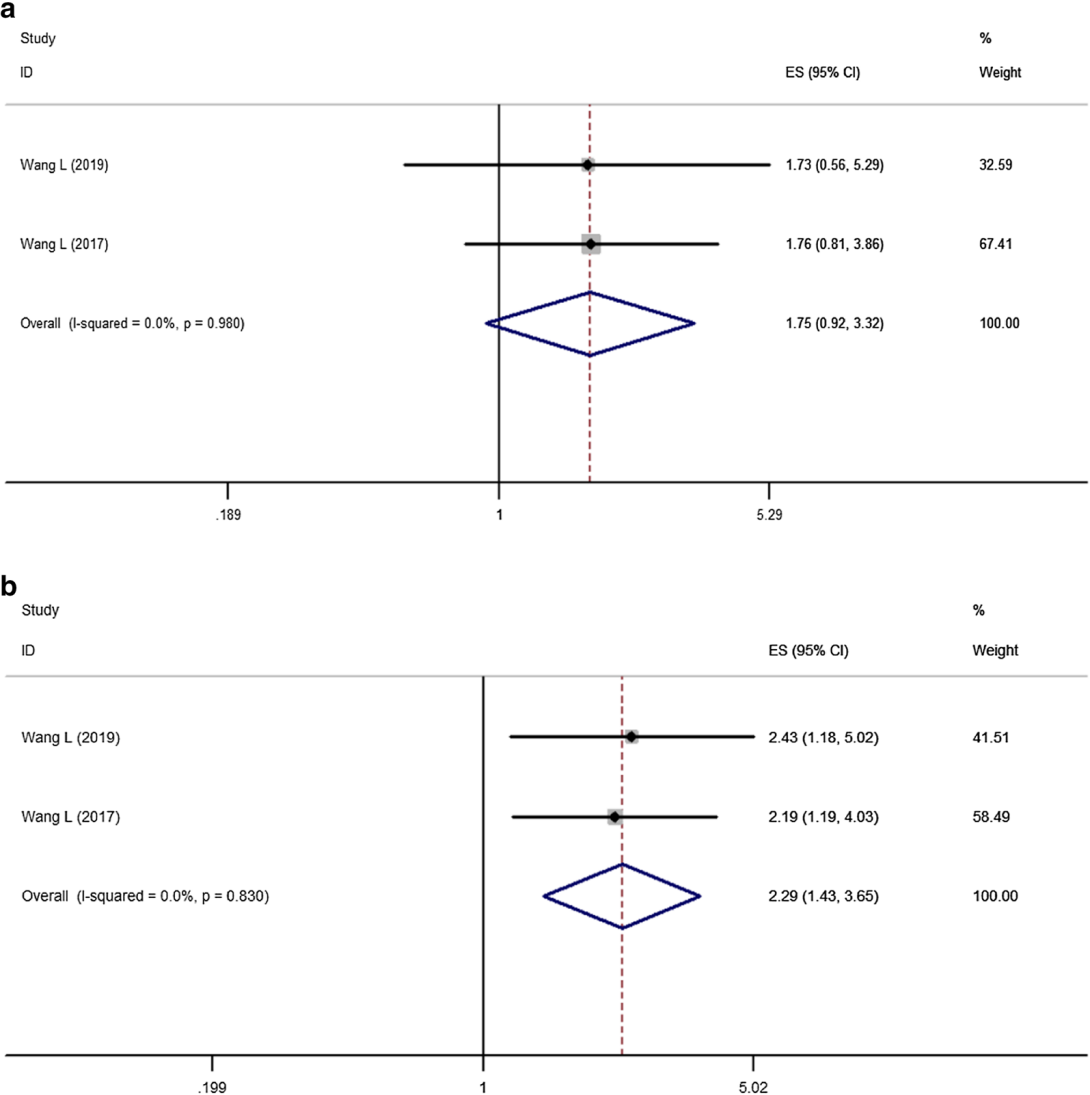
Forest plot of the overall survival and recurrence-free survival rates between postoperative TACE and radiotherapy. **a** Overall survival; **b** recurrence-free survival

Two retrospective studies [12, 13] compared the effects of pa-TACE and radiotherapy on OS in patients with HCC with MVI after curative resection. A total of 186 patients were included in the two studies, all of whom were Child–Pugh A. No significant differences were found between the two groups (HR: 1.75, 95% CI: 0.92–3.32, p = 0.087, Fig. 6a).

### Different RFS between postoperative radiotherapy and pa-TACE in patients with MVI

Two retrospective studies [12, 13] compared the effects of pa-TACE and radiotherapy on OS in patients with HCC with MVI after curative resection. A total of 186 patients were enrolled in these studies, all of whom were Child–Pugh A. The results showed that the effect of pa-TACE on RFS of patients with HCC with MVI was worse than that of postoperative adjuvant radiotherapy (HR: 2.29, 95% CI: 1.43–3.65, p < 0.001, Fig. 6b).

### The different effects of pa-TACE and re-resection/radiofrequency ablation on OS

Two retrospective studies [14, 18] compared the effects of pa-TACE and RF/re-resection on OS in patients with HCC with MVI after curative resection. A total of 129 patients were enrolled in these two studies. These patients with HCC with MVI experienced recurrence after curative resection. The results showed that there was no significant difference between pa-TACE and RF/re-resection in OS (HR: 0.65, 95% CI: 0.09–4.89, p = 0.671).

### Different OS and RFS between postoperative conservative treatment and adjuvant treatments in patients with MVI

**Fig. 7:**
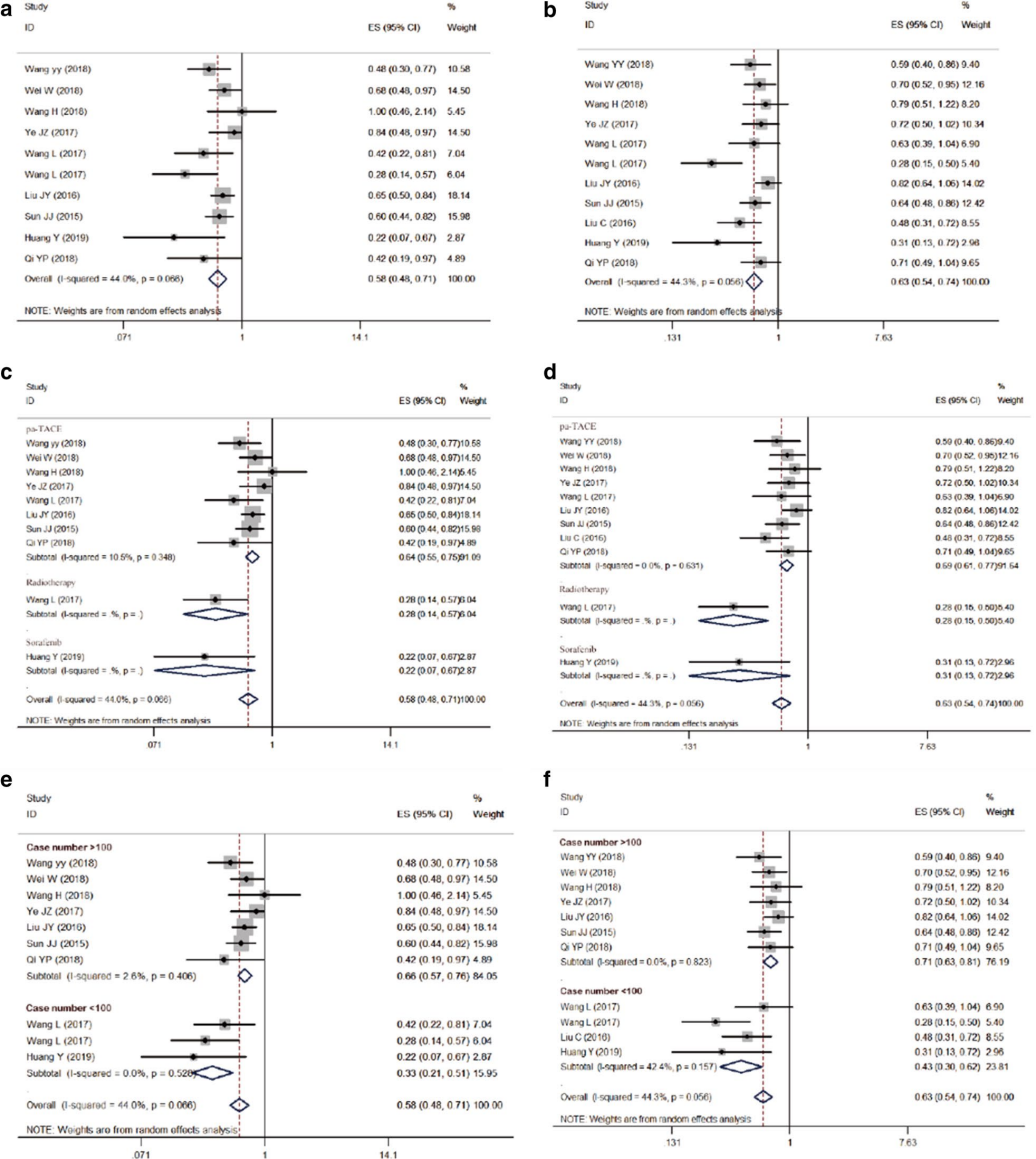
Forest plot of the overall survival and recurrence-free survival rates between postoperative adjuvant treatments and conservation. **a** Overall survival; **b** recurrence-free survival; **c** subgroup analysis of OS between different postoperative adjuvant treatments and conservation; **d** subgroup analysis of RFS between different postoperative adjuvant treatments and conservation. **e** subgroup analysis of OS based on case number; **f** subgroup analysis of DFS based on case number

Because few studies [13, 23] have compared OS and RFS between sorafenib/radiotherapy and conservative treatment in patients with MVI after curative resection, we combined pa-TACE, radiotherapy and sorafenib as adjuvant treatments. A total of eleven studies [11, 13, 15–20, 22, 23] compared the OS and RFS of adjuvant treatments and postoperative conservation treatment. The results showed that adjuvant treatments could significantly improve the OS and RFS of patients compared with the postoperative conservative group (HR: 0.580, 95% CI: 0.480–0.710, p < 0.001; HR: 0.630, 95% CI: 0.540–0.740, p < 0.001, retrospectively). Meanwhile, we further analyzed the influence of different treatments on the results. The pooled HR of pa-TACE was 0.640 (95% CI: 0.550–0.750, p < 0.001) for OS and 0.690 (95% CI: 0.610–0.770, p < 0.001) for RFS. The pooled HR of radiotherapy was 0.280 (95% CI: 0.140–0.570, p < 0.001) for OS and 0.280 (95% CI: 0.150–0.500, p < 0.001) for RFS. The pooled HR of sorafenib was 0.220 (95% CI: 0.070–0.710, p < 0.001) for OS and 0.310 (95% CI: 0.130–0.720, p < 0.001) for RFS. According to the subgroup analysis of case number, the pooled HR of case number less than 100 was 0.330 (95% CI: 0.210–0.510, p < 0.001) in OS and 0.430 (95% CI: 0.540–0.740, p < 0.001) in RFS. The pooled HR of case number greater than 100 was 0.660 (95% CI: 0.570–0.760, p < 0.001) for OS and 0.710 (95% CI: 0.630–0.810, p < 0.001) for RFS. The results of each subgroup were consistent with the overall results. All results are exhibited in Fig. 7.

## Discussion

Microvascular invasion (MVI) refers to the microscopic appearance of hepatocellular carcinoma cells in the portal vein or vascular lumen covered by endothelial cells adjacent to tumor tissue [5]. The major detection method is postoperative pathological examination; until now, there has been no effective preoperative diagnosis method. The latest study [8] showed that 38.7% of patients with HCC who received preoperative regular antiviral treatment were combined with MVI, and 51.9% of patients without preoperative regular antiviral treatment were combined with MVI. As one of the key factors affecting the recurrence and survival of patients with HCC, MVI has a poor prognosis in patients who only receive simple radical resection. The recurrence rate of patients with HCC with MVI was more than 20% [37], and the five-year survival rate was just 24% [9]. The fact that there is no effective way to diagnose MVI before surgery, the inability to detect it by the naked eye during surgery and the unclear effect of postoperative adjuvant therapy have hindered the development of treatment for patients with HCC with MVI.

This is the first systematic review and meta-analysis to evaluate the effect of various adjuvant therapies on patients with HCC with MVI after curative resection. The results showed that compared with postoperative conservative treatment, pa-TACE, postoperative radiotherapy and sorafenib could significantly improve the OS and RFS of such patients; the effect of pa-TACE on the RFS of patients with HCC with MVI was worse than that of postoperative adjuvant radiotherapy. Therefore, pa-TACE, radiotherapy and sorafenib can be superiorly used to improve the prognosis of patients with HCC combined with MVI after curative resection. Postoperative radiotherapy was better than pa-TACE in the treatment of patients with HCC with MVI.

Postoperative adjuvant transcatheter arterial chemoembolization (pa-TACE) is an adjuvant therapy that is commonly used after HCC curative resection. Pa-TACE can effectively prevent postoperative recurrence in patients with risk factors for recurrence, such as portal vein cancer thromboembolism [38]. By analyzing eight enrolled studies [11, 13, 15–19, 21], we found that pa-TACE was superior to postoperative conservative treatment in improving the prognosis of patients with HCC with MVI after curative resection. We further analyzed the effects of three strategies of pa-TACE (fluorouracil combined with doxorubicin, platinum and doxorubicin). The results indicated that these three pa-TACE strategies could all improve the prognosis of such patients, but there was a lack of studies comparing the effects among these three strategies. This suggested that pa-TACE was beneficial to patients with HCC with MVI after curative resection in clinical practice, which could not only reduce postoperative recurrence but also prolong the survival time of patients. Specific pa-TACE strategies should be formulated in consideration of patients’ physical tolerance and economic conditions. The evidence was not enough to prove that pa-TACE could improve the prognosis of patients with Child–Pugh B HCC with MVI after curative resection. We need more high-level studies to support these ideas.

Radiotherapy is a local treatment method. Compared with surgical treatment, radiotherapy has less trauma and can minimize the pain of patients. Currently, it has been widely used for nasopharyngeal cancer and esophageal cancer [39, 40]. In the past, radiotherapy was not widely applied in the treatment of HCC because of poor equipment, which might cause great injury to the liver. With the development of technology and the upgrading of equipment, an increasing number of studies have found that radiotherapy can not only kill the tumor but also prevent serious injury to the normal tissues around the tumor, reduce the recurrence rate and prolong the survival time [41–45]. We analyzed the current literature on postoperative adjuvant radiotherapy of patients with HCC with MVI after curative resection [13] and found that postoperative adjuvant radiotherapy can reduce patients’ recurrence rate and prolong survival time compared with postoperative conservative treatment. We further compared the efficacy of pa-TACE and radiotherapy in patients with HCC with MVI after curative resection [12, 13]. Although there was no obvious difference between the above two postoperative adjuvant treatments on OS, radiotherapy was significantly better than pa-TACE in preventing recurrence. Considering that the adverse effects of radiotherapy are smaller than pa-TACE, radiotherapy can bring larger benefits to these patients when the physical and economic conditions of patients permit. However, because the number of cases in the studies was not large enough and they were all retrospective studies, the evidence level was not high. Therefore, we need more high-level RCT study evidence to support our views.

Sorafenib is a kinase inhibitor. On the one hand, it can inhibit the RAF/MEK/ERK signal transduction pathway directly and prevent tumor growth. On the other hand, it can cut off the tumor cell nutrition supply and inhibit tumor growth through the inhibition of vascular endothelial growth factor receptors (VEGFR-1, VEGFR-2, VEGFR-3), platelet-derived growth factor receptor (PDGFR-β) and the formation of tumor angiogenesis [46]. Sorafenib was approved by the FDA in 2007 as a first-line treatment for HCC [47]. Several studies have shown that sorafenib could effectively prevent recurrence of HCC and prolong patient survival time [31, 48–51]. W We analyzed related studies [23] and found that sorafenib could significantly improve the OS and RFS of patients with HCC with MVI after curative resection compared with postoperative conservative treatment. However, in the choice of adjuvant therapy, there was no direct evidence to compare the effectiveness of TACE, radiotherapy and sorafenib. Tamai et al. [50] indicated in 2017 that sorafenib combined with TACE was not better than TACE alone in patients with unresectable HCC. Further studies are needed to determine whether sorafenib combined with TACE has greater benefits in patients with HCC with MVI after curative resection.

For patients with recurrent HCC with MVI after curative resection, the therapeutic effect of RF/re-resection was not significantly different from that of TACE. However, due to the small number of included studies and large heterogeneity among them, the level of evidence for this result was low, and more high-quality studies need to be analyzed to reach a clear conclusion. Because of the complexity of recurrent HCC, treatment strategies should be developed in combination with the tumor location and number of recurrent liver lesions, the patient’s liver condition, complications and the level of expertise of the treatment center.

In conclusion, it is necessary to conduct large-scale randomized prospective studies on the efficacy of various adjuvant therapies in patients with HCC with MVI after curative resection. We need more options for postoperative adjuvant therapies and a high level of evidence to guide clinical work.

## Limitations

This study had the following limitations: 1, all the included studies were from China; 2, only one RCT was included in this study; 3, there was a lack of horizontal comparison of various adjuvant therapeutic effects; and 4, there is heterogeneity among the studies, such as differences in surgical details, differences in the duration and dosage of various treatments and differences in the medical level of each center.

## Conclusion

Pa-TACE, radiotherapy and sorafenib can improve the prognosis of patients with HCC with MVI after curative resection compared with postoperative conservative treatment. Radiotherapy is superior to pa-TACE in preventing recurrence in patients with HCC with MVI after curative resection. More randomized controlled trials are needed to verify these conclusions.

## Data Availability

All data analysed and presented in this study are available from the corresponding author on reasonable request.
